# Binding antibody titers against the hemagglutinin and neuraminidase correlate with protection against medically attended influenza A and B disease

**DOI:** 10.1128/jvi.00391-25

**Published:** 2025-05-13

**Authors:** Marios Koutsakos, Arnold Reynaldi, Malet Aban, Ian G. Barr, David S. Khoury, Miles P. Davenport, Ali H. Ellebedy, Philip A. Mudd

**Affiliations:** 1 Department of Microbiology and Immunology, The University of Melbourne at the Peter Doherty Institute for Infection and Immunity, Melbourne, Victoria, Australia; 2 Kirby Institute, University of New South Wales, Kensington, New South Wales, Australia; 3 WHO Collaborating Centre for Reference and Research on Influenza, Royal Melbourne Hospital, at the Peter Doherty Institute for Infection and Immunity, Melbourne, Victoria, Australia; 4 Department of Pathology and Immunology, Washington University School of Medicine 12275 , St. Louis, Missouri, USA; 5 Center for Vaccines and Immunity to Microbial Pathogens, Washington University School of Medicine, St. Louis, Missouri, USA; 6 The Andrew M. and Jane M. Bursky Center for Human Immunology & Immunotherapy Programs, Washington University School of Medicine, St. Louis, Missouri, USA; 7 Department of Emergency Medicine, Washington University School of Medicine, St. Louis, Missouri, USA; St. Jude Children's Research Hospital, Memphis, Tennessee, USA

**Keywords:** influenza, hemagglutinin, neuraminidase, antibody, correlates of protection

## Abstract

Human challenge and cohort studies have identified various correlates of protection (CoP) against influenza A and B viruses (IAV/IBV). However, associations with viral load, investigation of mucosal CoPs, and CoPs against IBV are limited in the context of natural infections. Plasma and nasal swabs were collected (2017–2020) from 56 adults diagnosed with IAV (*n* = 25 H1N1, *n* = 19 H3N2) or IBV (*n* = 9 B/Victoria, *n* = 3 B/Yamagata) in the emergency department. Viral load was determined in nasal swabs. Antibodies (total Ig and IgA) specific for the hemagglutinin (HA) and neuraminidase (NA) of contemporary viruses from the subtype or lineage infecting each individual were measured by enzyme-linked immunosorbent assay (ELISA). Antibodies to a non-infecting influenza strain were measured and used as “control cases” to determine associations with protection from infection. Viral load decreased with time post-symptom onset. The Ct value at which 50% of the samples were positive in viral culture was 24.75 (95% confidence intervals, 23.7–27.01). Systemic HA and NA-specific Ig titers correlated with protection from medically-attended influenza disease. Neither systemic nor mucosal antibody measurements were associated with disease severity. We observed an inverse correlation between Ig anti-NA antibodies in nasal swabs and viral load by Ct value (regression coefficient = 3.25, CI = 0.3–6.2, *P* = 0.031), though this analysis was not corrected for multiple comparisons. Overall, high titers of HA and NA-specific antibodies measured by ELISA were associated with protection from the development of influenza A or B disease. Further work is needed to understand immune parameters associated with viral clearance and mucosal CoPs.

**IMPORTANCE:**

There is a great need to better understand correlates of protection (CoP) against influenza A and B viruses (IAV/IBV). In our study, we analyzed paired plasma and nasal swabs from patients presenting with influenza A or B disease as well as control patients. We measured hemagglutinin (HA) and neuraminidase (NA) specific antibodies in both sample types and also determined the amount of virus in nasal swabs. We found that higher systemic binding antibodies to the hemagglutinin and neuraminidase were associated with protection from medically attended disease. These findings expand our understanding of correlates of protection against influenza viruses and identify areas of future research to further understand protection from influenza.

## INTRODUCTION

Current influenza vaccines are only moderately effective, and further work is needed to improve the protection engendered by influenza vaccination. Understanding protective mechanisms and establishing correlates of protection (CoPs) in human cohorts is paramount in such endeavors. Systemic hemagglutination inhibition (HAI) titers, a surrogate for neutralizing antibodies, are a well-established correlate of protection against infection (1, 2). However, it is also appreciated that individuals with low HAI titers can be protected from infection and that additional correlates of protection other than HAI exist (1, 3). The establishment of CoPs needs to consider the context of infection (natural or controlled human challenge), functional versus binding antibodies and their targets, systemic versus mucosal measurements, as well as clinical endpoints (protection against infection, protection against severe disease, or reduction in viral shedding).

Antibodies as a CoP against influenza have been established in controlled human challenge studies. High pre-challenge serum HAI titers have been associated with protection from the acquisition of infection (typically determined by viral shedding) with H3N2 influenza A virus (IAV) (4, 5), H1N1pdm09 IAV (6, 7), or influenza B virus (IBV) (5), as well as with shorter duration of viral shedding and fewer symptoms when infection occurs (6). High pre-challenge serum neuraminidase inhibition (NAI) titers have also been associated with protection from the establishment of infection (viral shedding) with H3N2 or H1N1 IAV (4), as well as shorter duration of viral shedding of H3N2 IAV (8, 9) or H1N1pdm09 IAV (6, 7) and shorter duration of symptoms after infection (6). In studies where antibody measurements are based on enzyme-linked immunosorbent assay (ELISA) and not functional antibodies (HAI/NAI), high serum IgG titers against H1 have been associated with decreased viral shedding (7), while high serum IgA titers against H1 have been associated with fewer days of upper respiratory viral culture positivity (10). While these studies report systemic antibodies as a CoP, a limited number of studies have shown that higher pre-challenge nasal IgG and IgA titers against H3 or H1 IAV are associated with protection from infection (4) or decreases in the duration of upper respiratory viral culture positivity (10).

Antibodies as a CoP have also been investigated in naturally acquired influenza infection, usually identified by reverse transcription-PCR (RT-PCR) or seroconversion of individuals with influenza-like illness. Protection from infection with H3N2 or H1N1pdm09 is associated with high pre-infection HAI titers in serum (11–15) or nasal secretions (11), and with high NAI titers (11, 15) or anti-NA ELISA titers (12) in serum. Serum HAI and NAI are considered independent correlates of protection (11, 15). High serum microneutralization titers have also been associated with protection from infection (14, 15), as have high anti-HA stalk ELISA titers (12, 16, 17), the latter considered independent of HAI (12, 17). Elevated NA-specific ELISA titers in serum, but not HA stalk-specific antibodies or HAI, have also been associated with decreased duration of viral shedding of H1N1pdm09 (18). However, very little investigation has been performed evaluating antibody titer associations with nasal viral loads, looking at mucosal antibody titers as a CoP, and evaluating CoPs against IBV in the context of natural infections.

Here, in a cohort of medically attended adults naturally infected with IAV or IBV, we assessed the relationship between systemic and mucosal ELISA antibodies against the HA or NA at the time of diagnosis with viral load as well as with protection from the development of medically attended influenza disease or from severe disease.

## MATERIALS AND METHODS

### Participants and sample collection

We analyzed paired plasma and nasopharyngeal (NP) swab samples from the EDFLU study, which has been previously described (19, 20). Briefly, patients who had a clinical influenza real-time reverse-transcription polymerase chain reaction test (Cepheid Xpert Flu/RSV) performed during the normal course of workup and evaluation in the emergency department and were diagnosed with influenza A or B were approached. EDFLU study inclusion criteria required that subjects actively experience influenza-like illness symptoms at some point in the 24 hours before enrollment. Informed consent was obtained from all subjects or their legally authorized representatives. Peripheral blood samples were obtained from enrolled subjects into EDTA-anticoagulated tubes (BD Biosciences), and plasma was frozen at −80°C. NP swab samples were also obtained at enrollment for viral load analysis. NP swabs were collected using Becton Dickinson swabs (Becton Dickinson, New Jersey, USA) in 3 mL of universal virus transport media by trained nurse clinical research coordinators using standard clinical methods. Following collection, NP swabs were placed in universal transport media, transported to the laboratory, vortexed vigorously for 1 minute, and aliquots were stored at −80°C until further analysis.

For this study, 56 samples (Table S8) were selected based on the availability of sufficient volumes of both plasma and NP swabs for antibody analysis, as well as viral load analysis in NP swabs and IAV subtype/IBV lineage determination. Viral nucleic acid from NP swabs was used to determine the IAV subtype and IBV lineage. Samples were collected in the 2017/2018, 2018/2019, or 2019/2020 influenza seasons.

Days post-symptom onset were determined by research coordinators directly asking subjects at the point of study enrollment how long their illness symptoms had lasted. Progression of disease was measured both at the point of study entry (admission to hospital or discharged to home) and by retrospective review of the enrolled participants’ charts in the days/weeks following enrollment to determine the hospital length of stay, requirements for oxygen, requirements for ICU care, requirements for endotracheal intubation, and other outcomes during any hospital stay for those subjects not discharged home from the emergency department (ED). We classified illness severity in our influenza-infected cohort based on measured peripheral blood oxygen saturation using pulse oximetry. This classification strategy has been used previously by us and others (19–21). We classified as having “severe” illness any subject who presented with measured oxygen saturation ≤93% on room air, or who required supplemental oxygen. The category of “very severe” illness encompassed subjects who required tracheal intubation and mechanical ventilation owing to hypoxic respiratory failure. We established a “not severe” illness group to control for significant variation in clinical attributes of study subjects who did not meet criteria for severe or very severe illness. The subjects in the “not severe” group met all of the following criteria: (i) oxygen saturation ≥98% on room air, (ii) age <65 years, (iii) a chest radiograph was obtained during normal clinical care and read by the attending radiologist as clear, (iv) a medical history that did not include any of the Advisory Committee on Immunization Practices—specified risk factors for severe influenza illness (22), and (v) discharge from the hospital after a stay of <48 hours from the time of presentation to the hospital. In addition, some individuals were initially admitted to the hospital by the ED team, but almost immediately discharged by the inpatient medical team. In these cases, all lasting less than 48 hours after the patient entered the door of the ED, it did not seem appropriate to include those patients in the severe group if they had a clear chest x-ray, oxygen saturation ≥98% on room air, and no risk factors for severe influenza illness as defined by the Advisory Committee on Immunization Practices (ACIP). Therefore, to create the best comparisons of immune responses between severe and non-severe individuals, we used risk factors such as individuals >65 years of age or other ACIP-defined risk factors for severe illness to exclude individuals without respiratory failure from the control non-severe group who had a higher risk of having delayed recognition of true severe disease. Antiviral and NSAID treatment were not recorded for subjects in the study as all samples were collected in the ED prior to or in tandem with the initiation of medications and would not have a substantial bearing on the outcomes evaluated (hypoxic respiratory failure vs non-severe illness) in the time frame these outcomes were evaluated.

### Assessment of antibodies by ELISA

ELISAs were performed as previously described (23). Briefly, 96-well plates (MaxiSorp; Thermo Fisher, MA, USA) were coated with 100 µL of antigen diluted to 1 µg mL^−1^ in PBS by incubating overnight at 4°C. The following antigens were used: influenza virus H1 and N1 (A/Brisbane/2/2018; Sino Biological, Beijing, China), influenza virus H3 and N2 (A/Hong Kong/4801/2014; kindly provided by Florian Krammer), influenza virus B HA and NA (B/Washington/2/2019 and B/Phuket/3703/2013; Sino Biological) or bovine serum albumin (negative control). To determine the total amount of antibody plates that were coated with AffiniPure Goat Anti-Human IgA + IgG + IgM (H+L) (Jackson ImmunoResearch, PA, USA) at 1 µg mL^−1^ in PBS. Plates were blocked with 10% FBS and 0.05% Tween 20 in PBS. Plasma or NP swabs were serially diluted in blocking buffer and added to the plates. Plasma samples were diluted 1:50 and then threefold serially for the analysis of antigen-specific antibodies or 1:100 and then fivefold serially for the analysis of total antibody levels. NP swabs were diluted 1:3 and then twofold serially for the analysis of antigen-specific antibodies or 1:20 and then fivefold serially for the analysis of total antibody levels. Plates were incubated for 90 minutes at room temperature and then washed three times with 0.05% Tween 20 in PBS. Secondary antibodies conjugated to horseradish peroxidase (HRP) were diluted in blocking buffer before being added to wells and incubating for 60 minutes at room temperature. The following secondary antibodies were used: goat anti-human IgG (H+L)-HRP (goat polyclonal against IgG heavy chain and Ig light chains, Jackson ImmunoResearch, 1:2,500), goat anti-human IgG (polyclonal, Fcγ fragment specific, 1:1,500, Jackson ImmunoResearch), goat anti-human IgA (polyclonal, Jackson ImmunoResearch, 1:2,500). Plates were washed three times with 0.05% Tween 20 in PBS and three times with PBS before the addition of o-phenylenediamine dihydrochloride peroxidase substrate (Sigma-Aldrich). Reactions were stopped by the addition of 1 M hydrochloric acid. Optical density measurements were recorded at 490 nm. For each experiment, purified IgG or IgA monoclonal antibodies produced in-house as previously described (24) were used to generate standard curves from which concentrations were interpolated using a sigmoid 4PL curve in GraphPad Prism v9. As the goat anti-human IgG (H+L)-HRP reacts with both the heavy and the light chain, we consider detection with this antibody to reflect the total immunoglobulin (Ig) present for the ELISA antigen, and not a specific subtype.

### Real-time RT-PCR for the detection and characterization of the influenza virus

Multiplex real-time assay (one-step RT-PCR) was used to determine influenza in viral RNA extracted from nasopharyngeal swabs (140 µL) using QIAamp Viral RNA Mini Kit (Qiagen) as per the manufacturer’s instructions. The assay was based on the method by the National Influenza Centre, Public Health England, UK, as described in the World Health Organization Information for the Molecular Detection of Influenza viruses, February 2021. The 25 µL reaction mixture contained 6.25 µL of 4× Taqpath 1-Step Multiplex Master Mix (No Rox) (Thermofisher Scientific), 4 µL of primer mix (40 µM), 1.3 µL of probe mix (10 µM), 8.45 µL of nuclease-free water, and 5 µL of viral RNA. Thermocycling conditions consisted of 2 minutes at 25°C (UNG incubation), 15 minutes at 50°C (reverse transcription), 2 minutes at 95°C (polymerase activation), 45 cycles of 15 seconds at 95°C and 30 seconds at 55°C (PCR amplification). A specimen is considered positive for influenza A/H1pdm09, A/H3, or B when the amplification curve of the specific target crosses the threshold line within 40 cycles. Samples that were negative or influenza B positive in the multiplex real-time assay were further tested in a singleplex real-time one-step RT-PCR assay using primers and probes from the Centers for Disease Control and Prevention, Influenza Division, Atlanta, USA (available at the Influenza Reagent Resource [http://www.influenzareagentresource.org/]). Each 20 µL reaction contained 10 µL of 2× SensiFAST Probe Lo-ROX Mix (Bioline), 0.2 µL each of forward and reverse primers (40 µM), 0.2 µL of probe (10 µM), 0.2 µL of reverse transcriptase (RT/Taq), 0.4 µL of RNase inhibitor, 4.8 µL of nuclease-free water, and 4 µL of viral RNA. Thermocycling conditions consisted of 10 minutes at 45°C (reverse transcription), 2 minutes at 95°C (polymerase activation), 40 cycles of 5 seconds at 95°C, and 30 seconds at 60°C (PCR amplification). A specimen is considered positive for influenza A/H1pdm09, A/H3, B/Victoria, or B/Yamagata when the amplification curve of the specific target crosses the threshold line within 36 cycles. All real-time assays were carried out using the Applied Biosystems 7500 Real Time PCR System.

### Determination of tissue culture infectious dose

The infectious virus titer of the nasopharyngeal swabs was determined using the mean tissue culture infectious dose assay (TCID_50_) with ELISA readout (WHO Global Influenza Surveillance Network Manual for the laboratory diagnosis and virological surveillance of influenza, 2011). Samples were serially diluted (1/2 log_10_ starting from 1:10 and 1:20, four replicates each) in a sterile 96-well flat bottom plate (Greiner Bio-One Cellstar) in DMEM (Gibco) supplemented with 25 mM Hepes (Sigma), 100 U/mL, 100 mg/mL Penicillin/Streptomycin solution (Sigma), 1% bovine serum albumin, and 0.002 mg/mL TPCK-Trypsin (Worthington Biochem), leaving the last well for each replicate virus free as the cell control. The virus dilutions were then incubated at 37°C with 5% CO_2_. After 1 hour, MDCK-SIAT cells at 1.5 × 104 cells/well were added and incubated for 18–20 hours at 37°C with 5% CO_2_. Prior to staining, the inoculum was removed, plates washed with 1× PBS, and fixed in 80% cold acetone for 10–12 minutes at room temperature. After fixing, acetone was removed, plates were air-dried, then washed 3× with 0.3% Tween 20 (Sigma). To each well, 100 µL diluted primary antibody (mouse anti-Type A NP at 1:1,000 for influenza A or mouse anti-Type B NP at 1:10,000 for influenza B viruses) in blocking buffer (5% wt/vol skim milk powder in 0.3% Tween 20) was added and incubated for 1 hour at room temperature. Plates were washed 3× with 0.3% Tween 20 prior to the addition of 100 µL per well of secondary antibody (goat anti-mouse IgG-HRP conjugate-BioRad) diluted 1:1,000 in blocking buffer and incubated for 1 hour at room temperature. Unbound antibodies were removed by washing 5× with 0.3% Tween 20. Then, 100 µL of TMB substrate (Seracare) was added to each well and incubated for 10 minutes at room temperature in the dark. To stop the reaction, 100 µL of 1 M hydrochloric acid was added to all wells, and absorbance was read at 450 nm (OD450). Any test well with an OD450 greater than 2× the mean OD450 of the cell control wells is scored as positive. The TCID_50_ of each virus was calculated using the Reed-Muench method.

### Statistical analysis

A univariate regression model was used to assess the significance of age, days post-onset, and antibody levels on viral load measurement (as Ct values and culture positive). A logistic link function was used when running a model with a binary outcome (culture positive). Then, a multivariate model was used to assess the relationship of the variables (age, days post-onset, and various antibody levels) simultaneously against viral load. When predicting the infection status, the model is


$$Infection\;status=\alpha_{1}Age\;+\alpha_{2}DaysPostOnset\;+\alpha_{3}HA\;Ig\;plasma+\alpha_{4}HA\;Ig\;plasma+\alpha_{5}HA\;IgA\;plasma+\alpha_{6}NA\;IgA\;plasma.$$

Throughout this study, we reported the regression coefficient (quantifying the magnitude of the predictor variables against infection status) and the associated *P*-value (if this predictor is predictive of infection status). The significance of the regression coefficient was determined by Wald’s test, which can be obtained based on the standard error of the estimated parameters in the model. Variables for inclusion in the final model were selected by performing both forward and backward selection and selecting the model with the lowest Akaike Information Criterion. *P*-values of <0.05 were considered significant. The model was fitted using the *glmm and stepAIC* libraries in R.

## RESULTS

### Clinical cohort

To understand how systemic and mucosal antibodies specific for HA and NA correlate with protection from the development of clinical disease, protection from severe disease, or the magnitude of viral load, we analyzed paired plasma and nasal swab samples from the EDFLU study (19). We collated samples from 56 patients collected during the 2017/2018, 2018/2019, and 2019/2020 influenza seasons in St. Louis, Missouri, USA, based on sufficient sample availability for analysis of antibodies and to confirm IAV subtype or IBV lineage, using RT-PCR from the nasal swabs. The 3 B/Yamagata-infected subjects were from the 2017/2018 season, and the 9 B/Victoria-infected subjects were from the 2019/2020 season. The 25 A/H1N1-infected subjects were from the 2018/2019 season (14/25) and the 2019/2020 season (11/25). The 19 A/H3N2-infected subjects were primarily from the 2017/2018 season (16/19), with the remaining 3/19 being from the 2018/2019 season. Cohort characteristics are listed in Table 1. Using a severity classification strategy previously applied by us and others (19–21), 38 subjects were classified as “not severe,” 14 as “severe," and 4 as “very severe.”

**TABLE 1:** Cohort characteristics

Characteristic	Value for:			
All cases	All (*n* = 56)	Not severe (***n*** = 38)	Severe (***n*** = 14)	Very severe (***n*** = 4)
% IAV (*n*)	79 (44)	74 (28)	86 (12)	100 (4)
% IBV (*n*)	21 (12)	26 (10)	14 (2)	0 (0)
Age, mean (IQR), *y*	47 (29–61)	41 (27–58)	63 (52–72)	50 (46–54)
% Female sex (*n*)	55 (31)	60 (23)	50 (7)	25 (1)
Days post-symptom onset, mean (IQR), *d*	3 (2–4)	3 (1.75–4)	3 (1.75–4)	4.5 (1.75–6.5)
% Vaccinated in study season (*n*)				
No	59 (33)	68 (26)	36 (5)	50 (2)
Yes	32 (18)	24 (9)	64 (9)	0
Unknown	9 (5)	8 (3)	0	50 (2)

IAV	All (*n* = 44)	Not severe (*n* = 28)	Severe (*n* = 12)	Very severe (*n* = 4)
% H1N1 (*n*)	57 (25)	50 (14)	58 (7)	100 (4)
% H3N2 (*n*)	43 (19)	50 (14)	41.6 (5)	0
Age, mean (IQR), *y*	50 (32–63)	43 (27–60)	65 (53–73)	50 (46–54)
% Female sex (*n*)	55 (24)	60 (17)	50 (6)	25 (1)
Days post-symptom onset, mean (IQR), *d*	3 (1.25–4)	3 (1.25–4)	2.5 (1.25–4)	4.5 (1.75–6.5)
% Vaccinated in study season (*n*)				
No	59 (26)	71 (20)	33 (4)	50 (2)
Yes	34 (15)	25 (7)	67 (8)	0
Unknown	6.8 (3)	3.6 (1)	0 (0)	50 (2)

IB	All (*n* = 12)	Not severe (*n* = 10)	Severe (*n* = 2)	Very severe (*n* = 0)
% B/Yam (*n*)	25 (3)	20 (2)	50 (1)	
% B/Vic (*n*)	75 (9)	80 (8)	50 (1)	
Age, mean (IQR), *y*	37 (26–49)	34 (25–38)	52 (43–60)	
% Female sex (*n*)	58.3 (7)	60 (6)	50 (1)	
Days post-symptom onset, mean (IQR)	3.5 (2–4)	2.5 (1.75–4.5)	4	
% Vaccinated in study season (*n*)				
No	58.3 (7)	60 (6)	50 (1)	
Yes	25 (3)	20 (2)	50 (1)	
Unknown	16.7 (2)	20 (2)	0	

For each plasma and nasal swab sample, we determined antibody levels to HA and NA for total Ig and specifically for IgA by ELISA. We focused our analyses on binding antibodies due to the lack of established protocols for measuring HAI in mucosal samples. The HA and NA antigens used were from vaccine strains of the study seasons that were available commercially, although we note that we do not know the extent to which they are antigenically matched to the infecting strain of each participant. Given that we previously observed high variability in the amount of antibodies detectable in nasal swabs, antigen-specific antibody levels were normalized to the total amount of Ig or IgA in each sample to account for variability in nasal swab collection (23). These antibody measurements, where antigens were matched to the infecting IAV subtype or IBV lineage and which we refer to as “Flu-positive,” were used to assess relationships between antibodies and viral load or disease severity. To assess whether any antibody measurements correlate with protection from the development of medically attended influenza disease, we measured antibodies against a non-infecting influenza subtype and used those as “control cases” for the non-infecting influenza subtype. Specifically, some samples from IAV-infected subjects (based on availability) were tested against IBV antigens to generate a control data set for IBV-infected subjects. Similarly, some samples from IBV-infected subjects were tested against IAV antigens to generate a control data set for IAV-infected subjects. The ages of the different groups were not significantly different (Table S1). Since this set of “control cases” comprises individuals with medically attended influenza-like illness, it can, at least in part, account for differences in medical care-seeking behavior.

### Nasal swab RNA viral load predicts culture positivity

**Fig 1:**
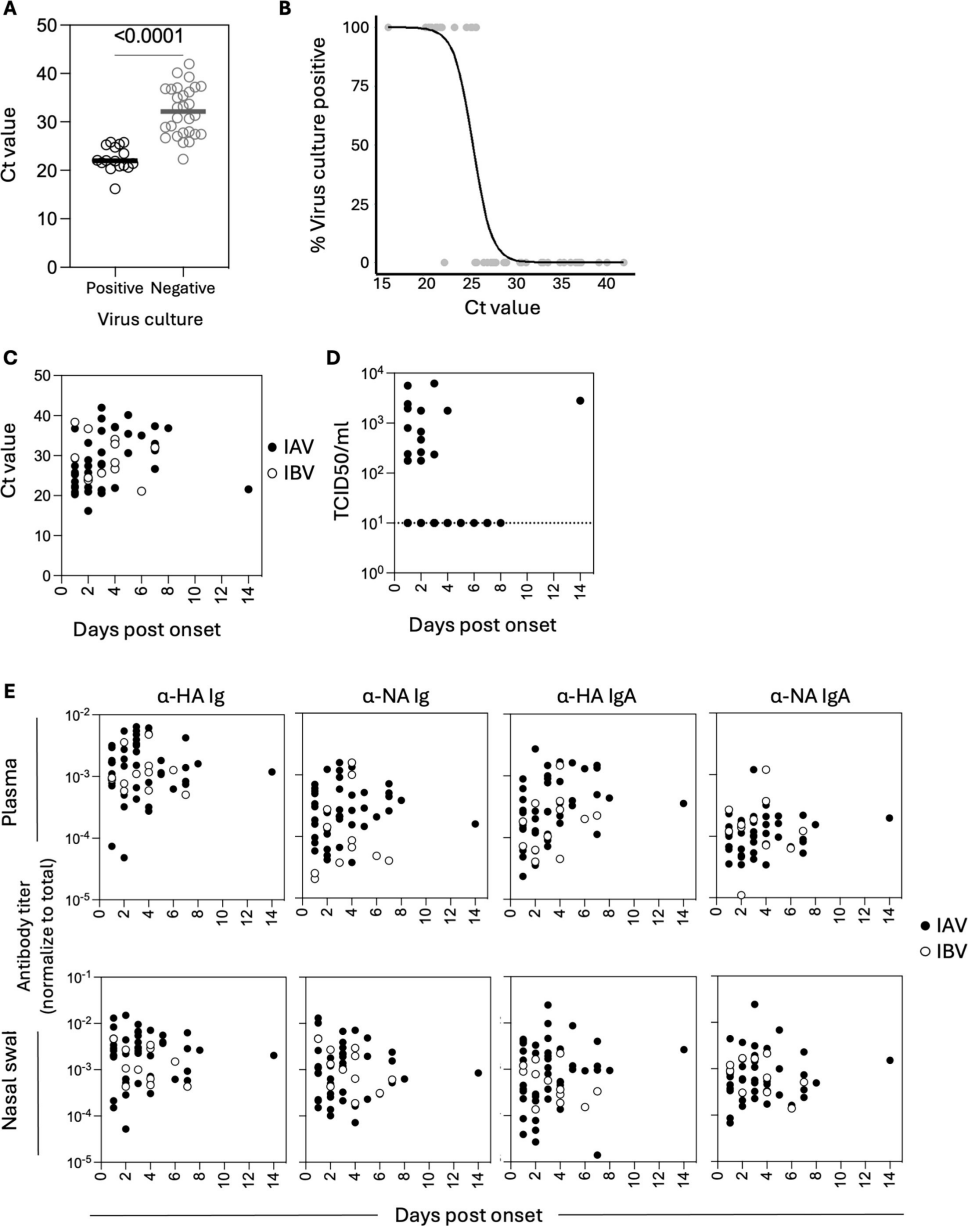
Viral load and antibody kinetics. (**A**) Ct values in virus culture positive (*n* = 16) or negative (*n* = 28) nasal swabs. *P*-values were estimated using a Mann-Whitney test. (**B**) Logistic regression curve between Ct value and virus culture positivity. Only data for IAV^+^ subjects are included, as all IBV subjects were culture negative. (**C and D**) Kinetics of viral load based on Ct value (**C**) or TCID_50_. (**E**) Kinetics of HA and NA-specific Ig and IgA antibody titers in plasma and nasal swabs. Ig refers to the total immunoglobulin detected specific for each ELISA antigen.

Viral RNA load was determined using RT-PCR, and infectious viral load was determined using a TCID_50_ assay on MDCK cells. While 16/44 IAV nasal swabs were culture positive, only 1/12 IBV nasal swabs yielded infectious virus, and thus culture positivity was not further analyzed for IBV. The median Ct value of IAV samples that grew in culture (21.9; range 16.2–25.8) was lower compared to compared to samples that did not grow (median Ct 32.3; range 22.3–42; *P* < 0.001) (Fig. 1A). Using logistic regression, we determined that the Ct value at which 50% of the samples were positive in viral culture was 24.75 (95% confidence intervals = 23.7–27.01) (Fig. 1B). When considering how viral load varied between individuals sampled at different days post-symptom onset (Fig. 1C and D), neither Ct value nor TCID_50_ changed significantly, but we noted an individual with high viral load for both Ct value and TCID_50_ at 14 days post-symptom onset. As influenza viral load is usually cleared by day 10 post-infection (6, 9, 25, 26), this may be due to recall bias related to “days post-onset” being self-reported. When this individual was excluded, viral load decreased significantly with time for both Ct (linear regression slope 1.2 [95% CI 0.4–2.1]) and log_10_ TCID_50_ (linear regression slope −0.19 [95% CI −0.3 to −0.074]). When considering how systemic or mucosal HA and NA antibody levels varied between individuals sampled at different days post-symptom onset (Fig. 1E), we did not observe significant changes with days post-onset (linear regression of log_10_ titer not significantly different from 0), except for systemic HA-specific IgA titers (slope of log_10_ titers = 0.07 [95% CI 0.015–0.12]). We next assessed for correlations between systemic and nasal antibody measurements in paired samples. When considering all cases or IAV-positive cases, HA-specific Ig and IgA, as well as NA-specific Ig levels, were positively correlated between blood and nasal swabs (Table S2). This was only the case for HA-specific Ig when only considering IBV cases.

### Systemic HA and NA-specific antibody titers at time of diagnosis correlate with protection from medically attended influenza disease

**Fig 2:**
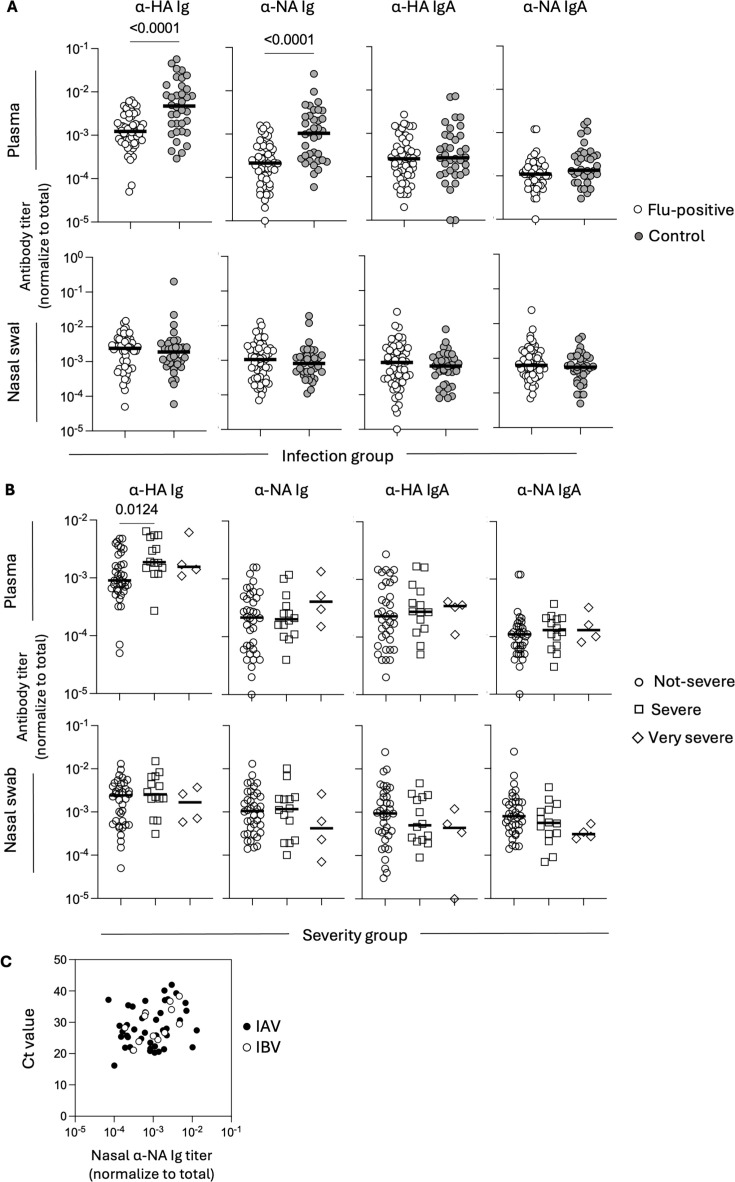
Systemic antibody titers correlate with protection from medically attended influenza disease. (**A**) HA- and NA-specific Ig and IgA antibody titers in plasma and nasal swabs between flu-positive cases (*n* = 56) and control samples (*n* = 48). *P*-values were estimated using a Mann-Whitney test. Ig refers to the total immunoglobulin detected specific for each ELISA antigen. (**B**) HA- and NA-specific Ig and IgA antibody titers in plasma and nasal swabs between flu-positive cases grouped by disease severity (non-severe *n* = 38, severe *n* = 14, very severe *n* = 4). *P*-values were estimated using a Kruskal-Wallis test with Dunn’s correction for multiple comparisons. (**C**) Correlation between nasal NA-specific Ig antibodies and Ct value. Throughout the figure, the interpolated ug/mL titer of each specificity was normalized to the total amount of Ig or IgA detected in each sample to account for variability in nasal swab collection.

When comparing the two infection groups, systemic Ig antibody titers to the HA and NA were higher in the control cases than in the “flu-positive cases” (Fig. 2A), suggesting an association between those two measurements and the development of medically attended influenza disease. No differences between the two groups were found in HA or NA IgA in plasma or in any of the nasal swab measurements (Fig. 2A). We further explored the association of antibodies with protection by fitting a mixed effects model with days post-onset and age as covariates and considering all flu cases, only IAV cases, or only IBV cases (Table S3). When all cases were included, HA and NA Ig and IgA measurements were higher in control cases than in flu-positive cases in plasma but not in nasal swabs. This result was the same when only IAV cases were included. When only IBV cases were included, HA and NA Ig, but not IgA, measurements were higher in control cases than in flu-positive cases in plasma but not in nasal swabs.

We further explored the association between HA and NA antibodies and protection, focusing on systemic measurements, as we did not observe any associations between nasal antibodies and protection. First, we confirmed these observed associations using μg/mL titers without normalizing to the total levels of Ig or IgA, as this was only relevant to nasal swabs, and obtained similar associations with protection (Table S4). Next, we tested the relative predictive value of different measurements concerning infection status (flu-positive or control case). To that end, we first fit a univariate model (considering the relationship of age, days post-onset, and each antibody measurement individually with infection status) and then a multivariate model (considering the relationship of age, days post-onset, and all antibody measurements simultaneously) (Table 2). The best multivariate model was obtained through a forward and backward model selection strategy (based on the Akaike Information Criterion), and both gave the same results. Interestingly, NA Ig measurements were the best predictor for IAV infection status, while for IBV, HA Ig was the best predictor of infection status. When considering all cases, the model, including both HA and NA Ig measurements, had the greatest predictive value. We note, however, that comparing the associations between HA or NA antibodies and protection may be confounded by a differential degree of antigenic similarity of the tested HA or NA antigens and the infecting strains, which we cannot determine in our study. Nonetheless, these data suggest that high antibody titers against both HA and NA may independently associate with protection from the development of medically attended disease.

**TABLE 2:** Predicting infection status by plasma antibody levels (μg/mL)^*a*^

	Univariate model		Best multivariate model	
	Regression coefficient	*P*-value	Regression coefficient	*P*-value
IAV cases				
Age (years)	0.011	0.47	**0.11^*b*^**	**0.012**
Days post-onset	0.07	0.58	–	–
HA Ig plasma	**−1.7**	**0.0087**	–	–
NA Ig plasma	**−4.44**	**0.00016**	**−7.35**	**0.00064**
HA IgA plasma	−0.66	0.25	**–**	**–**
NA IgA plasma	**−3.27**	**0.0016**	**–**	**–**
IBV cases				
Age (years)	0.012	0.59	–	–
Days post-onset	0.1	0.62	–	–
HA Ig plasma	**−3.24**	**0.011**	**−3.24**	**0.011**
NA Ig plasma	**−2.28**	**0.013**	–	–
HA IgA plasma	−0.41	0.48	–	–
NA IgA plasma	0.56	0.46	–	–
All cases				
Age (years)	0.016	0.16	**0.057**	**0.0011**
Days post-onset	0.077	0.45	–	–
HA Ig plasma	**−1.93**	**0.0001**	**−1.56**	**0.031**
NA Ig plasma	**−2.25**	**<0.0001**	**−2.05**	**0.0011**
HA IgA plasma	−0.08	0.82	–	–
NA IgA plasma	−0.73	0.15	–	–

^*a*^ Dashed lines indicate that the variable is not included in the best multivariate model, as determined by forward and backward selection.

^*b*^ Bold indicates variables with *P*-values <0.05.

Finally, we assessed for associations between systemic or nasal antibodies and disease severity or viral load. We did not find differences in systemic or mucosal antibodies to the HA or NA between severity groups, except for total anti-HA plasma antibodies in IAV-infected subjects, which were significantly higher in the very severe group (Fig. 2B; Table S5). Using multiple linear regression adjusting for age and days post-onset, we did not find an association between viral load (Ct value or TCID_50_) and systemic or mucosal antibodies in either IAV or IBV-infected subjects (Tables S6 and S7). When considering all subjects, we found an association between the levels of total Ig anti-NA antibodies in nasal swabs and viral Ct value (regression coefficient = 3.25, CI = 0.3–6.2, *P* = 0.031) (Fig. 2C), though we note we have not corrected for multiple comparisons in our analyses. Overall, we find that high titers of circulating, but not mucosal, antibodies to the HA as well as the NA are each associated with protection from the development of clinical IAV and IBV disease, but not disease severity or viral load.

## DISCUSSION

Although systemic HAI and NAI antibodies have been established as CoPs against infection with IAV, associations with viral load, investigations of mucosal CoPs, and CoPs against IBV are sparse in the context of natural infections. Here, we assessed anti-HA and anti-NA ELISA titers in blood and the nasal mucosa at the time of diagnosis in a cohort of medically attended adults naturally infected with IAV or IBV. Our data demonstrated that binding antibody titers against the HA and NA found in blood correlate with protection against medically attended influenza A and B disease.

Protection from infection has been typically associated with functional antibodies (HAI and NAI) in human challenge studies (4–7) and cohorts with natural infection (6, 11) (13–15). Recently, HA- and NA-specific ELISA titers have also been associated with protection from infection with H1N1pdm09 (12) and H3N2 (17). Our analyses further demonstrate an association between HA- and NA-specific ELISA titers and protection from the development of medically attended influenza disease, including IBV. Interestingly, mucosal binding antibodies were not associated with protection from medically attended disease. Further work is needed to explore associations between mucosal antibodies and protection using functional antibody measurements and in the context of milder and asymptomatic infections, neither of which are captured in our study. Nonetheless, our analyses of serum antibodies support an independent role for HA and NA antibodies in protection, reinforcing efforts to include NA antigens in influenza vaccines to increase the protection afforded by vaccination.

In addition to understanding the correlates of protection from infection, it is also important to understand the immune correlates of disease severity in individuals who become infected. In our analyses, we could not link systemic or mucosal binding antibodies to the HA or NA with disease severity, although the sample size of our study may have limited our power to detect such associations. It is possible that domain-specific antibodies (e.g., HA-stalk), functional antibodies (by neutralization or Fc-effector function), or measurements of cellular immunity would provide additional insights. It is also possible that antibodies in the upper respiratory tract are not reflective of immune responses in the lower respiratory tract, where pathology is often associated with severe disease. Additional studies are thus needed to determine mucosal correlates of protection from severe disease.

Previously, antibody levels against the HA and NA, including those at the mucosa, have been associated with reduced viral load and/or shedding duration (6–9, 18). Consistently, we observed a correlation between NA-specific antibodies in nasal swabs and viral load, although we iterate that our analysis was not powered to correct for multiple comparisons, and this observation needs to be verified in larger cohorts. A possible explanation for the lack of clear association between antibodies and viral load in our study is that our analysis is restricted to a single time point after infection and, therefore, does not capture the full dynamics of the immune response or viral replication kinetics. Alternatively, additional measurements of functional antibodies and cellular immunity may be required. Our results, therefore, do not preclude a role for systemic or mucosal antibodies as a correlate of viral load but rather suggest that longitudinal sampling, ideally including pre-infection samples, is likely required to fully understand the role of mucosal immunity in limiting viral replication in the context of natural infection.

There are several limitations to our study. First, the small sample size of patients infected with different subtypes may limit our power to detect small differences between groups. Second, as discussed above, the use of a single cross-sectional sample at the time of diagnosis may not be sufficient to fully understand the correlates of protection. Our analysis is based on a cohort of adults (29–61 years old), and associations with protection may differ in children or the elderly. The day post-symptom onset was based on self-reporting by the subjects, and we cannot determine the day of infection for each subject. Additionally, antiviral and NSAID treatment were not recorded, although they would not have a substantial bearing on the outcomes evaluated (hypoxic respiratory failure vs non-severe illness) in the time frame these outcomes were evaluated in. Finally, our analyses were specific for binding antibodies to the HA and NA, and we have not measured HAI, neutralizing antibodies, HA-stalk specific antibodies, or cellular immunity, systemically or at the mucosa, which may also contribute to reduced viral load and/or protection from the development of medically attended influenza disease. Despite these limitations, our analyses demonstrate a clear association between systemic HA and NA-specific antibody concentrations measured by ELISA and the development of medically-attended influenza A or B disease in a cohort of naturally infected individuals. Further work is needed to understand immune parameters that associate with viral clearance as well as mucosal CoPs.

## Data Availability

The data sets generated and/or analyzed during the current study are available from the corresponding author on reasonable request.

## SUPPLEMENTAL MATERIAL

Supplemental tables jvi.00391-25-s0001.pdf Tables S1 to S7.

Table S8 jvi.00391-25-s0002.xlsx Subject details.

ASM does not own the copyrights to Supplemental Material that may be linked to, or accessed through, an article. The authors have granted ASM a non-exclusive, world-wide license to publish the Supplemental Material files. Please contact the corresponding author directly for reuse.
