# Supplementary material for: Binding antibody titers against the hemagglutinin and neuraminidase correlate with protection against medically attended influenza A and B disease
Source: J Virol. 2025 May 13;99(6):e00391-25. doi: 10.1128/jvi.00391-25 (PMC12172496; doi:10.1128/jvi.00391-25)
Supplement: Supplemental tables — Tables S1 to S7. [file jvi.00391-25-s0001.pdf]

Supplementary Table 1. Age of influenza cases and control groups

	**Age, mean (IQR), y**	**p-value**
All flu-positive cases (n=56)	47 (29-61)	0.37
All control cases (n=36)	44 (28-59)	
IAV positive cases (n=44)	50 (32-63)	0.071
Control cases for IAV (n=18)	40 (27-53)	
IBV positive cases (n=12)	37 (26-49)	0.166
Control cases for IBV (n=18)	47 (32-60)	

Supplementary Table 2. Correlation between antibody levels in plasma and nasal swabs

		Spearman r	*p-value*
IAV only	**HA Ig**	**0.618**	**0.000**
	**NA Ig**	**0.454**	**0.002**
	**HA IgA**	**0.430**	**0.004**
	NA IgA	0.134	0.391
IBV only	**HA Ig**	**0.594**	**0.046**
	NA Ig	0.091	0.783
	HA IgA	0.217	0.499
	NA IgA	0.364	0.246
All cases	**HA Ig**	**0.621**	**0.000**
	**NA Ig**	**0.369**	**0.005**
	**HA IgA**	**0.437**	**0.001**
	NA IgA	0.190	0.165

Supplementary Table 3. Regression model of antibody levels (normalised to total) by infection status.

IAV cases

	**HA Ig Plasma**		**NA Ig Plasma**		**HA IgA Plasma**		**NA IgA Plasma**		**HA Ig Nasal**		**NA Ig Nasal**		**HA IgA Nasal**		**NA IgA Nasal**	
	coeff.	*p-val.*	*coeff.*	*p-val.*	*coeff.*	*p-val.*	*coeff.*	*p-val.*	*coeff.*	*p-val.*	*coeff.*	*p-val.*	*coeff.*	*p-val.*	*coeff.*	*p-val.*
(Intercept)	-3.490	0.000	-3.619	0.000	-4.266	0.000	-4.038	0.000	-3.391	0.000	-3.405	0.000	-3.472	0.000	-3.321	0.000
Age (years)	**0.019**	**0.004**	**0.014**	**0.001**	**0.011**	**0.042**	**0.008**	**0.035**	0.011	0.176	0.006	0.324	0.009	0.158	0.005	0.337
Days post-onset	0.044	0.413	**0.071**	**0.046**	**0.122**	**0.016**	0.033	0.293	0.032	0.644	0.030	0.554	-0.008	0.883	-0.036	0.433
Flu positive	**-0.618**	**0.0001**	**-0.978**	**0.0001**	**-0.377**	**0.002**	**-0.463**	**0.0001**	**-0.252**	**0.046**	**-0.341**	**0.038**	**-0.455**	**0.019**	-0.116	0.299

IBV cases

	**HA Ig Plasma**		**NA Ig Plasma**		**HA IgA Plasma**		**NA IgA Plasma**		**HA Ig Nasal**		**NA Ig Nasal**		**HA IgA Nasal**		**NA IgA Nasal**	
	coeff.	*p-val.*	*coeff.*	*p-val.*	*coeff.*	*p-val.*	*coeff.*	*p-val.*	*coeff.*	*p-val.*	*coeff.*	*p-val.*	*coeff.*	*p-val.*	*coeff.*	*p-val.*
(Intercept)	-1.98	0.00	-3.28	0.00	-3.21	0.00	-3.19	0.00	-3.03	0.00	-2.73	0.00	-3.57	0.00	-3.68	0.00
Age (years)	0.00	0.77	0.00	0.96	-0.02	0.08	-0.01	0.15	0.01	0.11	0.00	0.44	0.00	0.66	0.00	0.75
Days post-onset	-0.07	0.27	-0.01	0.91	0.13	0.19	0.08	0.20	-0.06	0.35	-0.06	0.31	0.10	0.14	0.07	0.27
Flu positive	**-0.66**	**0.01**	**-0.67**	**0.02**	-0.18	0.41	-0.18	0.31	-0.06	0.81	0.27	0.17	0.15	0.42	0.28	0.09

All cases

	**HA Ig Plasma**		**NA Ig Plasma**		**HA IgA Plasma**		**NA IgA Plasma**		**HA Ig Nasal**		**NA Ig Nasal**		**HA IgA Nasal**		**NA IgA Nasal**	
	coeff.	*p-val.*	*coeff.*	*p-val.*	*coeff.*	*p-val.*	*coeff.*	*p-val.*	*coeff.*	*p-val.*	*coeff.*	*p-val.*	*coeff.*	*p-val.*	*coeff.*	*p-val.*
(Intercept)	-2.850	0.000	-3.602	0.000	-3.631	0.000	-3.669	0.000	-3.248	0.000	-3.132	0.000	-3.522	0.000	-3.458	0.000
Age (years)	**0.010**	**0.034**	**0.011**	**0.020**	0.002	0.661	0.001	0.791	0.010	0.059	0.001	0.838	0.004	0.368	0.004	0.334
Days post-onset	0.008	0.847	0.041	0.305	**0.090**	**0.007**	**0.039**	**0.049**	0.006	0.905	0.000	0.995	0.037	0.361	0.002	0.965
Flu positive	**-0.660**	**0.0001**	**-0.799**	**0.0001**	**-0.207**	**0.018**	-0.122	0.077	-0.187	0.129	-0.119	0.349	-0.206	0.136	0.044	0.642

Supplementary Table 4. Regression model of plasma antibody levels (µg/ml) by infection status

IAV cases

	**HA Ig Plasma**		**NA Ig Plasma**		**HA IgA Plasma**		**NA IgA Plasma**	
	coeff.	*p-value*	*coeff.*	*p-value*	*coeff.*	*p-value*	*coeff.*	*p-value*
(Intercept)	1.047	0.0000	1.031	0.0000	-0.404	0.2922	-0.309	0.335
Age (years)	**0.014**	**0.0005**	**0.0070**	**0.049**	**0.014**	**0.03**	0.0072	0.181
Days post-onset	0.016	0.574	0.0289	0.291	0.078	0.1200	0.055	0.186
Flu positive	**-0.521**	**0.0004**	**-0.898**	**0.0000**	**-0.378**	**0.0012**	**-0.448**	**0.0001**

IBV cases

	**HA Ig Plasma**		**NA Ig Plasma**		**HA IgA Plasma**		**NA IgA Plasma**	
	coeff.	*p-value*	*coeff.*	*p-value*	*coeff.*	*p-value*	*coeff.*	*p-value*
(Intercept)	1.78	0	0.434	0.24	0.272	0.363	-0.0889	0.804
Age (years)	0.0051	0.381	0.0081	0.257	-0.0048	0.41	-0.0046	0.511
Days post-onset	-0.0063	0.902	0.046	0.469	**0.1412**	**0.0197**	0.084	0.197
Flu positive	**-0.668**	**0.0023**	**-0.751**	**0.0043**	-0.372	0.053	0.0715	0.617

All cases

	**HA Ig Plasma**		**NA Ig Plasma**		**HA IgA Plasma**		**NA IgA Plasma**	
	coeff.	*p-value*	*coeff.*	*p-value*	*coeff.*	*p-value*	*coeff.*	*p-value*
(Intercept)	1.308	0.000	0.616	0.001	-0.200	0.484	-0.239	0.323
Age (years)	**0.011**	**0.001**	**0.010**	**0.002**	**0.010**	**0.049**	0.003	0.426
Days post-onset	0.013	0.597	0.036	0.160	**0.087**	**0.032**	0.057	0.095
Flu positive	**-0.576**	**<0.0001**	**-0.708**	**<0.0001**	**-0.355**	**0.001**	**-0.273**	**0.007**

Supplementary Table 5. P-values from comparison of antibody levels (normalised to total) by severity group

	IAV	IBV	ALL
HA Ig Plasma	**0.038**	0.756	**0.030**
NA Ig Plasma	0.425	0.227	0.412
HA IgA Plasma	0.840	0.760	0.823
NA IgA Plasma	0.483	0.766	0.642
HA Ig Nasal	0.388	0.969	0.384
NA Ig Nasal	0.440	0.394	0.394
HA IgA Nasal	0.312	0.218	0.298
NA IgA Nasal	0.250	0.171	0.144

Supplementary Table 6. Regression model of viral RNA load by antibody levels (normalised to total)

IAV cases

	HA Ig Plasma		NA Ig Plasma		HA IgA Plasma		NA IgA Plasma		HA Ig Nasal		NA Ig Nasal		HA IgA Nasal		NA IgA Nasal	
	coeff.	*p-val.*	*coeff.*	*p-val.*	*coeff.*	*p-val.*	*coeff.*	*p-val.*	*coeff.*	*p-val.*	*coeff.*	*p-val.*	*coeff.*	*p-val.*	*coeff.*	*p-val.*
(Intercept)	20.79	0.02	25.97	0.01	31.19	0.00	7.28	0.01	22.05	0.00	29.68	0.00	20.43	0.00	19.25	0.00
Antibody level	-0.24	0.92	1.11	0.64	2.15	0.30	-3.56	0.27	0.14	0.94	2.40	0.16	-0.35	0.79	-0.78	0.68
Age (years)	0.10	0.09	0.09	0.09	0.07	0.20	0.10	0.07	0.09	0.08	0.08	0.11	0.10	0.06	0.09	0.07
Days post-onset	0.68	0.07	0.66	0.08	0.53	0.18	0.75	0.05	0.68	0.07	0.69	0.06	0.69	0.07	0.69	0.07

IBV cases

	HA Ig Plasma		NA Ig Plasma		HA IgA Plasma		NA IgA Plasma		HA Ig Nasal		NA Ig Nasal		HA IgA Nasal		NA IgA Nasal	
	coeff.	*p-val.*	*coeff.*	*p-val.*	*coeff.*	*p-val.*	*coeff.*	*p-val.*	*coeff.*	*p-val.*	*coeff.*	*p-val.*	*coeff.*	*p-val.*	*coeff.*	*p-val.*
(Intercept)	30.62	0.00	21.64	0.00	42.79	0.00	31.96	0.01	34.48	0.01	45.99	0.01	58.92	0.00	48.61	0.01
Antibody level	1.06	0.91	-0.93	0.85	3.93	0.33	1.41	0.71	2.58	0.64	6.76	0.16	9.45	0.06	7.10	0.19
Age (years)	0.10	0.55	0.15	0.46	0.12	0.26	0.13	0.26	0.09	0.45	0.06	0.58	0.02	0.83	0.08	0.47
Days post-onset	-0.56	0.56	-0.47	0.66	-0.88	0.37	-0.56	0.56	-0.33	0.76	0.37	0.72	0.29	0.73	0.13	0.89

All cases

	HA Ig Plasma		NA Ig Plasma		HA IgA Plasma		NA IgA Plasma		HA Ig Nasal		NA Ig Nasal		HA IgA Nasal		NA IgA Nasal	
	coeff.	*p-val.*	*coeff.*	*p-val.*	*coeff.*	*p-val.*	*coeff.*	*p-val.*	*coeff.*	*p-val.*	*coeff.*	*p-val.*	*coeff.*	*p-val.*	*coeff.*	*p-val.*
(Intercept)	23.39	0.00	22.40	0.01	31.98	0.00	18.37	0.01	24.91	0.00	33.43	0.00	23.90	0.00	24.05	0.00
Antibody level	0.14	0.95	-0.12	0.95	2.06	0.25	-1.23	0.61	0.64	0.70	**3.25**	**0.03**	0.29	0.82	0.37	0.83
Age (years)	0.09	0.09	0.09	0.06	0.07	0.15	0.08	0.06	0.08	0.07	0.07	0.10	0.09	0.06	0.09	0.05
Days post-onset	0.53	0.12	0.53	0.12	0.38	0.29	0.54	0.11	0.53	0.12	0.59	0.07	0.52	0.13	0.53	0.12

Supplementary Table 7. Regression model of infectious viral load by antibody levels (normalised to total)

IAV cases

	**HA Ig Plasma**		**NA Ig Plasma**		**HA IgA Plasma**		**NA IgA Plasma**		**HA Ig Nasal**		**NA Ig Nasal**		**HA IgA Nasal**		**NA IgA Nasal**	
	coeff.	*p-val.*	*coeff.*	*p-val.*	*coeff.*	*p-val.*	*coeff.*	*p-val.*	*coeff.*	*p-val.*	*coeff.*	*p-val.*	*coeff.*	*p-val.*	*coeff.*	*p-val.*
(Intercept)	0.48	0.88	-0.62	0.86	-1.49	0.67	1.33	0.80	1.38	0.55	-0.11	0.96	1.96	0.31	2.21	0.32
Antibody level	-0.37	0.65	-0.61	0.48	-0.71	0.36	-0.06	0.96	-0.13	0.83	-0.58	0.34	0.05	0.92	0.13	0.84
Age (years)	-0.03	0.21	-0.03	0.14	-0.02	0.28	-0.03	0.15	-0.03	0.12	-0.03	0.14	-0.03	0.09	-0.03	0.10
Days post-onset	-0.27	0.12	-0.26	0.15	-0.21	0.27	-0.28	0.13	-0.28	0.12	-0.28	0.11	-0.28	0.12	-0.28	0.11
